# Elementary Lessons in Electricity

**Published:** 1879-02

**Authors:** A. Floyd Delafield


### ELEMENTARY LESSONS IN ELECTRICITY.

By A. FLOYD DELAFIELD, A.B.

It is my intention in these papers to draw up an outline of the principles of electricity, as concise as clearness permits, which shall afford to those using electrical apparatus sufficient information to enable them to select such apparatus with judgment, and apply it to the best advantage. In this number I shall explain some of the terms employed in the science, and describe a few experiments which illustrate the meaning and use of the terms. In subsequent numbers I shall describe the different forms of apparatus used for the production of electricity, and explain what results may be expected from each, giving particular attention to the selection and arrangement of batteries, and the different effects produced by differences in the number and size of their elements.

It is hoped that this series of articles will meet the wants of those who, while desiring exact and accurate information about electricity, cannot spare the time to study elaborate treatises.

I. Electricity is a form of energy. It cannot be defined, but is described by enumerating the phenomena it produces, such as attractions and repulsions, magnetic, chemical, physiological and other phenomena.

There are several ways of developing electricity.

*a.* By rubbing dissimilar substances together.

*b.* By placing dissimilar substances in contact or in a liquid.

*c.* By warming or cooling the junction of two dissimilar substances.

*d.* By changes in a system of magnets and wires, either in the strength of the magnets, or in the relative position of the parts of the system.

Besides these methods there are others not practically used.

The terms frictional electricity, galvanic electricity, magneto-electricity, etc., often employed, refer to electricity produced in different ways, and not to different kinds of electricity. We know of but one kind of electricity, whatever means may be employed to produce it.

2. A body having more or less electricity than the earth near it, is said to be electrified. If it has more than the earth, we say it is positively electrified, if less, negatively.

The degree of electrification of a body above or below the earth near it, irrespective of the size or nature of the body, is called its potential. It is analogous to temperature, which is also an idea independent of mass.

The course of differences of potential is called electromotive force. The unit or standard of electromotive force is called a volt, and is about equal to the electromotive force produced by any form of sulphate of copper battery. We say, for instance, the electromotive force of a certain battery is two volts, just as we should say the weight of an apple is six ounces, or the capacity of a tumbler is half a pint.

In any electrified body there is a certain quantity of electricity which can be measured.

The quantity of electricity which can by any means be gotten into a body is limited by its capacity, which depends on its surface—a hollow ball having the same capacity as a solid one.

The capacity of a body is affected by neighboring bodies.

3. If two bodies of different potentials be connected by a wire, the excess of electricity above the mean of the two passes from the body of higher to that of lower potential, and we say, a current of electricity passes from the first to the second.

When we speak of the strength of a current, we mean the quantity of electricity that passes per second.

It is found that the nature and size of the wire joining the two bodies of different potential, affect the strength of the current.

The degree to which the wire opposes the passage of the current is called its resistance, and is independent of the strength of the current.

Bodies of small resistance are called conductors. Those of very great resistance are called insulators.

The unit or standard of resistance is called an ohm, and is practically a certain piece of wire kept at Kew in London, exact copies of which have been sent all over the world, and are used for measuring the resistance of coils of wire, and for other purposes.

In measuring resistances we use a set of wires of one, two, etc., ohms resistance, just as in weighing we use a set of weights of one, two, etc., pounds or ounces.

[illegible]vo bodies at different potentials be connected by [illegible] having a very high resistance, such as air, [illegible]ia rubber, the potentials of the two bodies do not become equal; a certain quantity of electricity passes into the air or glass, or other insulator, and then the whole system attains a condition of equilibrium.

5. Electricity in equilibrium is statical electricity.

Electricity in motion is dynamical electricity.

A mass fixed at any height above the surface of the earth possesses energy similar to statical electricity.

A mass falling from such a height possesses energy similar to dynamical electricity.

II. Methods of producing electricity.

In treating of the methods of producing electricity, it is convenient to speak of friction, although for the production of electricity for practical purposes, this method is never, or at least seldom, used. There are certain very important experiments, however, which are most conveniently made with frictional apparatus. In making these, dry weather must be chosen, as a damp atmosphere is a conductor of electricity, and in such an atmosphere no great difference of potentials can be maintained.

The simplest form of frictional machine consists of a piece of glass, hard rubber, or sealing wax, and a piece of silk or a catskin. On rubbing one of the first mentioned bodies with one of the second, electricity is developed in both of them. The potential of one is raised and that of the other lowered.

If the electrified glass or other substance be held near some light body, this will be attracted to it, but as soon as it has touched the glass or other electrified body, it will be repelled.

This simple experiment is the basis of the whole science of electricity. It is most convenient to make it in the following way:

Provide three glass standards a few inches high, bent in the shape of a small Roman f, and fixed in wooden or metal bases. Let us call them nnmbers 1, 2 and 3.

Now from 1 and 2 suspend single small balls of pith by fine silk threads, so that they hang free, and from 3 suspend in the same way two balls so that they touch each other. Let us also have a rod or tube of glass a few inches long, a stick of ordinary sealing wax, and a piece of silk, such as a handkerchief. We can now make the following experiments:

1. Rub the glass or sealing-wax briskly with the silk handkerchief, then hold it to ball No. 1. The ball is attracted, but as soon as it has touched the rod, it is repelled.

2. Rub the rod again, and hold it then to the pair of balls No. 3. They behave as did the single ball, but on allowing them to touch the rubbed rod and then removing this, they will come to rest, not touching each other, however, but standing off from one another.

3. Bring 1 and 2 near each other. Touch No. 1 with the glass rod, after this has been rubbed, and touch No. 2 with the rubbed sealing-wax. The two balls will now attract one another, but if they be allowed to touch, they fall apart again, and lose all sign of electrification.

From these experiments we see that:—

1. An electrified substance attracts a light, non-electrified body.

2. An electrified substance repels a body that has touched it.

3. A body touched by rubbed glass or sealing-wax repels one touched by the same substance, and attracts one touched by the other substance.

It is by carefully making such experiments as these that the following laws have been demonstrated:

1. On rubbing glass and silk together, the potential of the glass is raised and that of the silk lowered.

2. On rubbing sealing-wax and silk together, the potential of the sealing-wax is lowered and that of the silk raised.

We know of no reason for this, any more than why electricity should be produced at all in either case. We can only say, such are the facts.

3. On bringing an electrified body in contact with one of a different potential, a portion of the electricity passes to the body of lower potential. It is for this reason that we can electrify the suspended balls by touching them with the rubbed rods of glass and sealing-wax. This also explains the fact that the two balls touched, one by glass of raised potential, and the other by sealing-wax of lowered potential, lose all sign of electrification on touching each other. The potential of one has been raised, for the glass touched it, electricity went into it—that of the other lowered, for electricity passed from it to the sealing-wax. Now, on bringing the balls together, supposing them to be of one size, all the electricity passes from the ball of higher potential to that of lower potential, and they are thus brought to the potential of the earth; that is, they are both at the potential zero—are not electrified at all.

4. Two substances, the potential of both of which is higher or lower than that of the earth, repel each other; if the potential of one be higher, and that of the other lower than that of the earth, they attract each other.

